# Xpert MTB/RIF Ultra versus Xpert MTB/RIF for the diagnosis of tuberculous meningitis: a prospective, randomised, diagnostic accuracy study

**DOI:** 10.1016/S1473-3099(19)30649-8

**Published:** 2020-03

**Authors:** Joseph Donovan, Do Dang Anh Thu, Nguyen Hoan Phu, Vu Thi Mong Dung, Tran Phu Quang, Ho Dang Trung Nghia, Pham Kieu Nguyet Oanh, Tran Bao Nhu, Nguyen Van Vinh Chau, Vu Thi Ngoc Ha, Vu Thi Ty Hang, Dong Huu Khanh Trinh, Ronald B Geskus, Le Van Tan, Nguyen Thuy Thuong Thuong, Guy E Thwaites

**Affiliations:** a Oxford University Clinical Research Unit, Centre for Tropical Medicine, Ho Chi Minh City, Vietnam; b Centre for Tropical Medicine and Global Health, Nuffield Department of Medicine, University of Oxford, Oxford, UK; c Viet Anh Ward, Hospital for Tropical Diseases, Ho Chi Minh City, Vietnam

## Abstract

**Background:**

Xpert MTB/RIF Ultra (Xpert Ultra) might have higher sensitivity than its predecessor, Xpert MTB/RIF (Xpert), but its role in tuberculous meningitis diagnosis is uncertain. We aimed to compare Xpert Ultra with Xpert for the diagnosis of tuberculous meningitis in HIV-uninfected and HIV-infected adults.

**Methods:**

In this prospective, randomised, diagnostic accuracy study, adults (≥16 years) with suspected tuberculous meningitis from a single centre in Vietnam were randomly assigned to cerebrospinal fluid testing by either Xpert Ultra or Xpert at baseline and, if treated for tuberculous meningitis, after 3–4 weeks of treatment. Test performance (sensitivity, specificity, and positive and negative predictive values) was calculated for Xpert Ultra and Xpert and compared against clinical and mycobacterial culture reference standards. Analyses were done for all patients and by HIV status.

**Findings:**

Between Oct 16, 2017, and Feb 10, 2019, 205 patients were randomly assigned to Xpert Ultra (n=103) or Xpert (n=102). The sensitivities of Xpert Ultra and Xpert for tuberculous meningitis diagnosis against a reference standard of definite, probable, and possible tuberculous meningitis were 47·2% (95% CI 34·4–60·3; 25 of 53 patients) for Xpert Ultra and 39·6% (27·6–53·1; 21 of 53) for Xpert (p=0·56); specificities were 100·0% (95% CI 92·0–100·0; 44 of 44) and 100·0% (92·6–100·0; 48 of 48), respectively. In HIV-negative patients, the sensitivity of Xpert Ultra was 38·9% (24·8–55·1; 14 of 36) versus 22·9% (12·1–39·0; eight of 35) by Xpert (p=0·23). In HIV co-infected patients, the sensitivities were 64·3% (38·8–83·7; nine of 14) for Xpert Ultra and 76·9% (49·7–91·8; ten of 13) for Xpert (p=0·77). Negative predictive values were 61·1% (49·6–71·5) for Xpert Ultra and 60·0% (49·0–70·0) for Xpert. Against a reference standard of mycobacterial culture, sensitivities were 90·9% (72·2–97·5; 20 of 22 patients) for Xpert Ultra and 81·8% (61·5–92·7; 18 of 22) for Xpert (p=0·66); specificities were 93·9% (85·4–97·6; 62 of 66) and 96·9% (89·5–91·2; 63 of 65), respectively. Six (22%) of 27 patients had a positive test by Xpert Ultra after 4 weeks of treatment versus two (9%) of 22 patients by Xpert.

**Interpretation:**

Xpert Ultra was not statistically superior to Xpert for the diagnosis of tuberculous meningitis in HIV-uninfected and HIV-infected adults. A negative Xpert Ultra or Xpert test does not rule out tuberculous meningitis. New diagnostic strategies are urgently required.

**Funding:**

Wellcome Trust and the Foundation for Innovative New Diagnostics.

## Introduction

*Mycobacterium tuberculosis* kills more people each year than any other infectious disease.^1^ Tuberculous meningitis is the most severe form of tuberculosis, resulting in death or disability in approximately half of those it affects.^2^ Delayed diagnosis and treatment are strongly linked to poor outcomes, a situation exacerbated by conventional diagnostic tests for *M tuberculosis*, which are insufficiently sensitive. Cerebrospinal fluid (CSF) smear microscopy is widely available, yet sensitivity following Ziehl–Neelsen staining is often low.^3^ Culture of *M tuberculosis* takes several weeks and cannot guide initial treatment decisions.

Xpert MTB/RIF (Xpert; Cepheid, Sunnyvale, CA, USA) offered a breakthrough in tuberculosis diagnostics: a rapid, highly sensitive nucleic acid amplification test (NAAT) with additional rifampicin susceptibility testing. Xpert uses a hemi-nested real-time PCR assay to detect and amplify an *M tuberculosis*-specific sequence of the bacterial *rpoB* gene.^4^ Xpert is valuable when positive, yet it is insufficiently sensitive to exclude tuberculous meningitis when negative. Meta-analyses of the diagnostic performance of Xpert for tuberculous meningitis showed pooled sensitivities of 79·5–80·5% compared with mycobacterial culture and specificities of 98·6–98·8% for *M tuberculosis* detection in CSF.^5^, ^6^ However, sensitivity is affected by the volume of CSF tested, whether CSF centrifugation was done before testing,^7^ and the choice of diagnostic gold standard. Use of a clinical reference standard, wherein not all cases are microbiologically confirmed, results in reduced Xpert sensitivity. Standardised diagnostic criteria proposed by Marais and colleagues^8^ are frequently used to compare tuberculous meningitis diagnostic test performance, yet inconsistency in inclusion of some or all of definite, probable, and possible cases in the reference standard limits study comparison.

**Research in context**

**Evidence before this study**

We searched PubMed Central for all studies or reports of Xpert MTB/RIF Ultra (Xpert Ultra) for the diagnosis of tuberculous meningitis, using the terms “tuberculous meningitis” OR “TB meningitis” OR “extrapulmonary” AND “Xpert Ultra”, up to Sept 14, 2019. No language restrictions were applied. Our search returned five relevant publications that included patients undergoing Xpert Ultra testing of cerebrospinal fluid (CSF) for diagnosis of tuberculous meningitis. One study did Xpert Ultra and Xpert testing on thawed pre-centrifuged cryopreserved CSF from 23 patients screened for a trial of treatment for HIV co-infected cryptococcal meningitis. Sensitivities were 69·6% (16 patients) for Xpert Ultra and 43·5% (ten patients) for Xpert, compared with a reference standard of definite or probable tuberculous meningitis. Of 21 cases positive by Xpert Ultra, Xpert Ultra was the only positive mycobacterial test in eight cases, suggesting Xpert Ultra might detect cases of tuberculous meningitis below the threshold of detection of other confirmatory mycobacterial tests. Subsequent to this study, WHO recommended Xpert Ultra replace Xpert in all settings.

Three studies (in four to 43 patients) compared Xpert Ultra and Xpert for diagnosis of extrapulmonary tuberculosis in patients with suspected tuberculous meningitis. The study containing the largest patient group used at least 3 mL of uncentrifuged CSF for testing. Sensitivities were 44·2% (19 of 43) for Xpert Ultra and 18·6% (eight of 43) for Xpert (p=0·01). Although the reported sensitivity of Xpert Ultra was higher than that of Xpert when testing bacteriologically confirmed tuberculous meningitis (86·4% [19 of 22] *vs* 36·4% [eight of 22]; p=0·001), cases positive only by nucleic acid amplification tests were included in the reference standard. Of the 43 cases, all were smear negative and only three were culture positive. A case series of 11 patients (two of whom were HIV co-infected) with definite or probable tuberculous meningitis who underwent CSF testing found positive tests in seven patients with Xpert Ultra and four with Xpert. No randomised comparison of the two methods has been done to date, and the role of Xpert Ultra in tuberculous meningitis diagnosis remains controversial.

**Added value of this study**

To our knowledge, this study in 205 individuals, including 108 treated for tuberculous meningitis, is the first randomised comparison of Xpert Ultra and Xpert. We provide information about the post-treatment performance of both tests. Tuberculous meningitis is associated with very low numbers of bacteria in CSF; therefore, the CSF volume tested is a crucial determinant of the diagnostic performance of tests that directly detect bacteria or their nucleic acids. We thus chose to collect and test large volumes (6 mL) of CSF and randomly assign patients to Xpert Ultra or Xpert CSF testing, rather than halving the CSF sample for use in two concurrent tests. This approach maximised the CSF volumes tested, providing a better estimate of diagnostic performance and mimicking clinical practice in which only one molecular test would normally be done. We showed that Xpert Ultra was not superior to Xpert when compared against either clinical or mycobacterial culture reference standards. Specificity of tuberculous meningitis diagnosis was not reduced with Xpert Ultra when compared against a clinical reference standard—an important finding, given the reduction in specificity previously described with Xpert Ultra testing of sputum samples for pulmonary tuberculosis. In pulmonary tuberculosis, Xpert Ultra has shown superior sensitivity to Xpert when testing sputum samples with low bacillary load. HIV-uninfected patients with tuberculous meningitis are considered to have lower CSF bacillary loads than those with HIV co-infection, and in the HIV-uninfected group of our study, Xpert Ultra had a higher sensitivity than Xpert against the clinical and mycobacterial culture reference standards, although the differences were not significant.

**Implications of all the available evidence**

Xpert Ultra showed a modest increase in sensitivity compared with Xpert for the diagnosis of tuberculous meningitis and after the start of antituberculosis treatment, but the differences were not significant. These differences appeared to be greater in HIV-uninfected than in HIV-infected patients with tuberculous meningitis, which suggests Xpert Ultra might perform better than Xpert when bacterial numbers are very low. However, Xpert Ultra's negative predictive value (61·1%) remains too low to be used to rule out tuberculous meningitis. The search must continue for a better diagnostic test for tuberculous meningitis.

Xpert MTB/RIF Ultra (Xpert Ultra) aims to improve the sensitivity of tuberculosis diagnosis and enhance rifampicin resistance identification. A larger reaction chamber, plus incorporation of two different multicopy amplification targets (IS*6110* and IS*1081*) intend to reduce the limit of detection of bacterial colony-forming units.^9^ Adaptation of molecular probes and testing approach are designed to differentiate between silent mutations and mutations conferring resistance.^10^ A prospective multicentre study in adults with pulmonary tuberculosis showed that Xpert Ultra had a higher diagnostic sensitivity than Xpert: 63% versus 46% in smear-negative, culture-positive sputum samples (n=135) and 90% versus 77% in culture-positive sputum samples from HIV co-infected individuals (n=115).^11^ However, no improvement in sensitivity was seen among HIV-uninfected individuals (91% for Xpert Ultra *vs* 90% for Xpert), and Xpert Ultra specificity was lower than Xpert (96% *vs* 98%).

In 2017, Bahr and colleagues^12^ reported the first published study of Xpert Ultra for the diagnosis of tuberculous meningitis, testing CSF obtained during screening for a study of HIV co-infected cryptococcal meningitis. In 23 HIV co-infected patients with definite or probable tuberculous meningitis, pre-centrifuged cryopreserved CSF was thawed and retrospectively tested with Xpert Ultra. In that study, the sensitivities were 69·6% (16 of 23) for Xpert Ultra and 43·5% (ten of 23) for Xpert when compared against a reference standard of definite or probable tuberculous meningitis. Subsequently, Wang and colleagues^13^ assessed Xpert Ultra for the diagnosis of extrapulmonary, paucibacillary tuberculosis, including 43 CSF samples from HIV-uninfected adults with suspected tuberculous meningitis. At least 3 mL of uncentrifuged CSF was tested by both Xpert Ultra and Xpert, with Xpert Ultra showing higher diagnostic sensitivity than Xpert (44·2% [19 of 43] *vs* 18·6% [eight of 43]; p=0·01) against a reference standard of definite, probable, and possible tuberculous meningitis. Smear or culture diagnoses were infrequent (none by smear and three by culture), and evaluation of sensitivity in bacteriologically confirmed cases relied heavily on cases positive only by Xpert Ultra or Xpert. Two other studies of Xpert Ultra testing of extrapulmonary samples included 16 and four CSF samples of suspected tuberculous meningitis, respectively,^14^, ^15^ of which two (13%) of 16 (both culture negative) and three (75%) of four (all culture positive) were positive by Xpert Ultra. In 2019, Chin and colleagues^16^ described 11 patients with definite or probable tuberculous meningitis (two of whom were HIV co-infected) who underwent CSF testing by both Xpert Ultra and Xpert. Tests were positive for seven (64%) patients with Xpert Ultra and four (36%) patients with Xpert.

To date, studies comparing Xpert Ultra with Xpert for tuberculous meningitis diagnosis have been small, involved retrospective testing, and have included few patients with microbiologically confirmed tuberculous meningitis. However, WHO now recommend replacement of Xpert with Xpert Ultra in all settings.^17^ We therefore did a large, randomised, prospective comparison of the two diagnostic tests to better define the role of Xpert Ultra in the diagnosis of tuberculous meningitis.

## Methods

### Study design and participants

We did a prospective, randomised, observational study to compare the performances of Xpert Ultra and Xpert for the diagnosis of tuberculous meningitis. In this study, diagnostic tests were randomly allocated to patients and diagnostic performances were evaluated; as this study was not a randomised clinical trial, it was not registered as such. Patients aged 16 years or older with suspected tuberculous meningitis based on clinical and CSF findings (clear or mildly cloudy CSF, plus >5 days of symptoms constistent with tuberculous meningitis^8^ or low CSF glucose or raised CSF lactate concentrations) at The Hospital for Tropical Diseases (Ho Chi Minh City, Vietnam) were eligible for enrolment. Patients were excluded if lumbar puncture was contraindicated or informed consent was not given by the patient or by a relative if the patient did not have capacity. The study was approved by the Hospital for Tropical Diseases and the Oxford Tropical Research Ethics Committee.

### Procedures

At baseline, patients were randomly assigned to undergo Xpert Ultra or Xpert testing of CSF obtained by lumbar puncture. A randomisation list was generated using a program written in R version 3.4. A CSF volume of 6 mL was used for mycobacterial tests; if less than 6 mL was taken, the tests were still done, with all CSF volumes recorded. CSF was centrifuged at 3000 g for 15 minutes. The supernatant was removed and the deposit was resuspended in the remaining 500 μL. 100 μL was used for Ziehl–Neelsen smear, 200 μL for mycobacterial culture (mycobacteria growth indicator tube [MGIT]), and 200 μL for either Xpert Ultra or Xpert. Ziehl–Neelsen smear, MGIT, and Xpert were done following standard procedures, as previously described.^18^ When MGIT testing was positive, phenotypic drug susceptibility testing was done by a Bactec MGIT SIRE kit (Becton, Dickinson; Franklin Lakes, NJ, USA) as previously described.^18^ Xpert Ultra and Xpert testing were done by laboratory technicians (DDAT, VTMD, TPQ) masked to the patient's clinical characteristics.

At the end of the trial, all patients received a final diagnosis of definite, probable, possible, or not tuberculous meningitis according to the published uniform case definition for tuberculous meningitis clinical research.^8^ Disease severity was assessed by the Medical Research Council tuberculous meningitis grade. Patients with probable and possible diagnoses could be reassigned to not tuberculous meningitis if the treating clinician did not consider the final diagnosis to be tuberculous meningitis and the patient recovered without antituberculosis chemotherapy. Patients were treated following local and national guidelines. Repeat testing was done according to the initial randomisation group on routine follow-up CSF taken 3–4 weeks after treatment initiation for those treated for tuberculous meningitis. Final reference standard diagnoses were assigned without the Xpert Ultra or Xpert result contributing to the final diagnosis.

HIV testing was not mandatory for this study and was done when clinically indicated. All patients reported as HIV negative had a negative HIV test at baseline. Of those reported HIV positive, either a test was positive at baseline or previous HIV positivity was recorded.

### Outcomes

Diagnostic performances (sensitivity, specificity, and positive and negative predictive values) of Xpert Ultra, Xpert, smear, and MGIT culture were compared against the clinical reference standards of definite, probable, and possible tuberculous meningitis, definite and probable tuberculous meningitis, and definite tuberculous meningitis. Additionally, diagnostic performances of Xpert Ultra and Xpert were compared against a mycobacterial reference standard (MGIT culture). Both clinical and microbiological reference standards were used because of the absence of a single gold-standard test for tuberculous meningitis. A post-hoc analysis of CSF volume influencing the likelihood of a positive Xpert Ultra or Xpert test was done, where CSF volume used for mycobacterial testing was divided into three categories: more than 5 mL, 2–5 mL, and no more than 2 mL, consistent with CSF volume intervals in a previous study.^18^ In addition, the diagnostic performances of Xpert Ultra and Xpert were evaluated by HIV status, given HIV co-infection has been shown to improve Xpert sensitivity for the diagnosis of tuberculous meningitis.^18^ Test performances after 3–4 weeks of antituberculosis treatment were also evaluated against the uniform case definition for tuberculous meningitis in patients who received antituberculosis chemotherapy. Diagnostic performances of Xpert Ultra and Xpert for rifampicin resistance prediction were evaluated against phenotypic drug-susceptibility testing of MGIT-positive cases. An exploratory analysis comparing Xpert and Xpert Ultra for semi-quantification of CSF bacterial numbers into high, medium, low, or very low categories (and trace for Xpert Ultra) was also done.

### Statistical analysis

Test performance measures (sensitivity, specificity, and positive and negative predictive values) with associated Wilson CIs were calculated for Xpert Ultra and Xpert and compared with those for Ziehl–Neelsen smear and MGIT using the χ^2^ test.

The study by Bahr and colleagues suggested Xpert Ultra was 25% more sensitive than Xpert for the diagnosis of tuberculous meningitis.^12^ Assuming the sensitivity of Xpert was 60%,^18^ using a significance level of 5% and 80% power, we calculated that 49 patients with tuberculous meningitis were required in each of the testing groups to be able to detect a 25% difference in sensitivity. To provide robust specificity estimates, CSF from at least 100 patients with non-tuberculous meningitis central nervous system infections was also tested.

Univariable and multivariable logistic regression analyses were done to identify factors associated with microbiological confirmation (ie, positive smear, Xpert, Xpert Ultra, or MGIT) of tuberculous meningitis. The following variables were tested: age, sex, duration of illness, Glasgow Coma Score, Medical Research Council tuberculous meningitis grade, CSF–blood glucose ratio, CSF lactate, CSF protein, CSF lymphocyte percentage, and CSF volume.

Statistical analysis was done using the programming language R (version 3.5.1).

### Role of the funding source

The funders had no role in study design, data collection, data analysis, data interpretation, writing of the report, or the decision to submit the paper for publication. The corresponding author had full access to all of the data in the study and had final responsibility for the decision to submit for publication.

## Results

**Figure 1:**
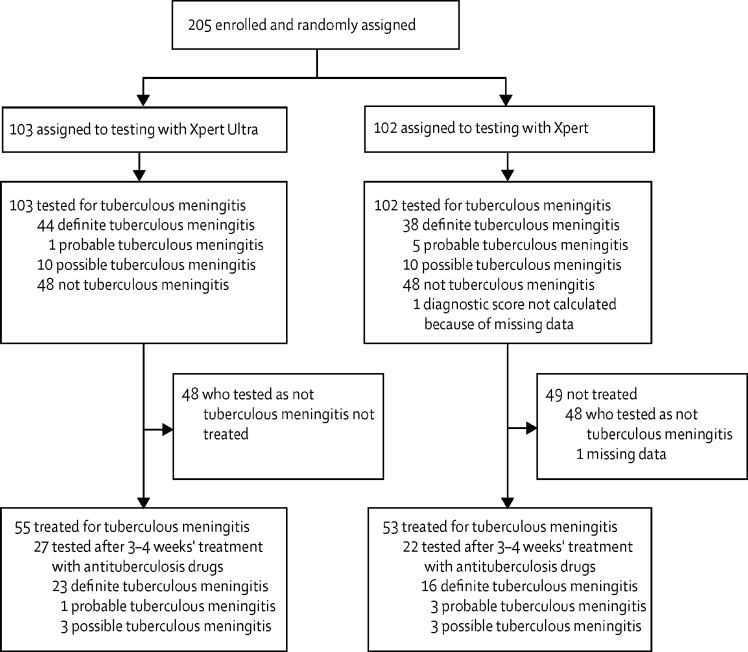
Trial profile Xpert Ultra=Xpert MTB/RIF Ultra. Xpert=Xpert MTB/RIF.

Between Oct 16, 2017, and Feb 10, 2019, 205 participants were consecutively enrolled into the study and randomly assigned to Xpert Ultra (n=103) or Xpert (n=102; figure 1). Of the 204 participants who obtained a final diagnosis, as per the uniform case definition for tuberculous meningitis,^8^ 82 (40%) were diagnosed with definite, six (3%) with probable, 20 (10%) with possible, and 96 (47%) with not tuberculous meningitis (figure 1; table 1). Baseline variables, including age and sex, in the Xpert Ultra and Xpert groups seemed well matched (table 1). Median CSF volumes used for mycobacterial testing were similar in both groups (table 1). Median time to MGIT positivity was 15 days (IQR 10–18) in the Xpert Ultra group and 18 days (13–20) in the Xpert group. Disease severity, HIV status, and CSF parameters also appeared well matched between the two groups (table 1).

**Table 1:** Baseline characteristics

		**Xpert Ultra (n=103)**	**Xpert (n=102)**
Age (years)		42 (31–57)	44 (33–58)
Sex			
	Female	41 (40%)	35 (34%)
	Male	62 (60%)	67 (66%)
Final diagnosis			
	Definite tuberculous meningitis	44 (43%)	38/101 (38%)
	Probable tuberculous meningitis	1 (1%)	5/101 (5%)
	Possible tuberculous meningitis	10 (10%)	10/101 (10%)
	Not tuberculous meningitis	48 (47%)	48/101 (48%)
Medical Research Council tuberculous meningitis grade*			
	1	19/55 (35%)	13/53 (25%)
	2	22/55 (40%)	25/53 (47%)
	3	14/55 (25%)	15/53 (28%)
HIV status			
	Positive	17 (17%)	14/99 (14%)
	Negative	48 (47%)	48/99 (48%)
	Unknown	38 (37%)	37/99 (37%)
CSF characteristics*			
	CSF white cell count (per μL)	310 (172–597);n=55	334 (120–484); n=53
	CSF lymphocytes	76% (38–88);n=55	74% (41–86);n=52
	CSF protein (g/L)	1·87 (1·12–2·79); n=55	1·96 (1·31–2·92);n=53
	CSF–blood glucose ratio	0·35 (0·22–0·46); n=55	0·35 (0·25–0·43); n=53
	CSF volume for mycobacterial tests (mL)	5·8 (5·0–6·0);n=100	5·5 (5·0–6·0);n=101
	Time to CSF MGIT positivity (days)†	15 (10–18);n=23	18 (13–20);n=22

Data are n (%), n/N (%), or median (IQR); number assessed is listed with the median when not assessed in all patients. Xpert Ultra=Xpert MTB/RIF Ultra. Xpert=Xpert MTB/RIF. CSF=cerebrospinal fluid. MGIT=mycobacteria growth indicator tube.

* Medical Research Council grade and CSF characteristics shown only for 108 patients with definite, probable, or possible tuberculous meningitis, with the exception of CSF volume for mycobacterial tests.

† Data shown for all 45 CSF samples for which MGIT was positive.

The diagnostic sensitivities of Xpert Ultra and Xpert against the reference standard of definite, probable, and possible tuberculous meningitis were 47·2% (95% CI 34·4–60·3) for Xpert Ultra and 39·6% (27·6–53·1) for Xpert (p=0·56; table 2). Specificities of Xpert Ultra and Xpert were both 100·0% (table 2). Against a mycobacterial culture reference standard, sensitivities were 90·9% (95% CI 72·2–97·5; 20 of 22) for Xpert Ultra and 81·8% (61·6–92·7; 18 of 22) for Xpert and specificities were 93·9% (85·4–97·6; 62 of 66) for Xpert Ultra and 96·9% (89·5–99·2; 63 of 65) for Xpert. The sensitivities of Xpert Ultra and Xpert were similar against reference standards of definite and probable tuberculous meningitis (Xpert Ultra *vs* Xpert p=0·52) and definite tuberculous meningitis (p=0·87). Sensitivities were 59·5% (44·5–73·0; 25 of 42) for Xpert Ultra and 55·3% (39·7–69·9; 21 of 38) for Xpert against the reference standard of definite tuberculous meningitis (p=0·87; table 2). Neither Xpert Ultra nor Xpert was as sensitive as Ziehl–Neelsen smear against any reference standard (table 2). Ziehl–Neelsen smear was significantly more sensitive than both Xpert Ultra and Xpert (data not shown), as shown in previous papers.^3^, ^18^

**Table 2:** Diagnostic performance of Xpert Ultra and Xpert against clinical reference standard

	**Xpert Ultra**	**Xpert**	**Ziehl–Neelsen smear**	**MGIT culture**
**Reference standard: definite, probable, and possible tuberculous meningitis**				
Positive tests	25/53*	21/53	77/108	45/94
Sensitivity	47·2% (34·4–60·3)	39·6% (27·6–53·1)	71·3% (62·5–79·0)	47·9% (38·0–57·9)
Specificity	100·0% (92·0–100·0)	100·0% (92·6–100·0)	100·0% (96·1–100·0)	100·0% (95·6–100·0)
PPV	100·0% (86·7–100·0)	100·0% (84·5–100·0)	100·0% (95·2–100·0)	100·0% (92·1–100·0)
NPV	61·1% (49·6–71·5)	60·0% (49·0–70·0)	72·2% (67·2–82·1)	63·2% (54·7–70·9)
**Reference standard: definite and probable tuberculous meningitis**				
Positive tests	25/43	21/43	77/88	45/75
Sensitivity	58·1% (43·3–71·6)	48·8% (34·6–63·2)	87·5% (79·0–92·9)	60·0% (48·7–70·3)
Specificity	100·0% (93·4–100·0)	100·0% (93·8–100·0)	100·0% (96·8–100·0)	100·0% (96·4–100·0)
PPV	100·0% (86·7–100·0)	100·0% (84·5–100·0)	100·0% (95·2–100·0)	100·0% (92·1–100·0)
NPV	75·0% (63·9–83·6)	72·5% (61·9–81·1)	91·3% (85·0–95·1)	77·4% (69·6–83·7)
**Reference standard: definite tuberculous meningitis**				
Positive tests	25/42	21/38	77/82	45/73
Sensitivity	59·5% (44·5–73·0)	55·3% (39·7–69·9)	93·9% (86·5–97·4)	61·6% (50·2–72·0)
Specificity	100·0% (93·5–100·0)	100·0% (94·3–100·0)	100·0% (96·3–100·0)	100·0 (96·5–100·0)
PPV	100·0% (86·7–100·0)	100·0% (84·5–100·0)	100·0% (95·2–100·0)	100·0% (92·1–100.0)
NPV	76·4% (65·4–84·7)	78·8% (68·6–86·3)	95·3% (89·4–98·0)	79·0% (71·3–85·0)

Data in parentheses are 95% CIs. Xpert Ultra=Xpert MTB/RIF Ultra. Xpert=Xpert MTB/RIF. MGIT=mycobacteria growth indicator tube. PPV=positive predictive value. NPV=negative predictive value.

* Of 55 cases of definite, probable, or possible tuberculous meningitis tested by Xpert Ultra, two with definite tuberculosis returned an error result. Therefore, only 53 cases are included in the sensitivity calculation. Of the 205 participants enrolled in the study, Ziehl–Neelsen smear and MGIT were done in 204 (>99%) and 187 (91%) cases, respectively.

**Figure 2:**
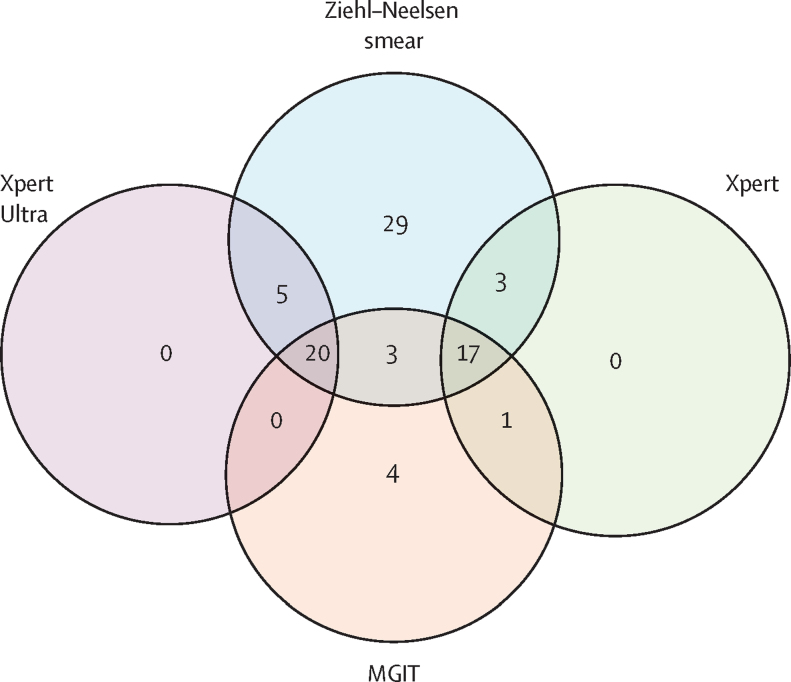
Positive mycobacterial tests in individuals with at least one confirmatory test for tuberculous meningitis 168 positive mycobacterial tests (25 positive by Xpert Ultra, 21 by Xpert, 77 by Ziehl–Neelsen smear, and 45 by MGIT) from 82 patients with a diagnosis of definite tuberculous meningitis. MGIT=mycobacteria growth indicator tube. Xpert Ultra=Xpert MTB/RIF Ultra. Xpert=Xpert MTB/RIF.

When considering the distribution and overlap of positive CSF by Xpert Ultra, Xpert, Ziehl–Neelsen smear, and MGIT, all positive Xpert Ultra or Xpert cases were also positive by Ziehl–Neelsen smear, MGIT, or both (figure 2). There were six error results with Xpert Ultra and none with Xpert. Eight MGIT samples showed contaminated growth. Tested CSF volumes did not vary substantially (median 5·8 mL, IQR 5·0–6·0, in all patients combined; table 1), but CSF volume did not appear to influence the likelihood of a positive Xpert Ultra or Xpert test (appendix p 1). Univariable and multivariable analysis of factors predicting microbiological confirmation of tuberculous meningitis are shown in the appendix (p 2). Female sex was associated with a reduced likelihood of microbiological confirmation of tuberculous meningitis in both univariable and multivariable analyses, with all other factors non-predictive in the multivariable analysis.

HIV testing was done in 127 (63%) of 202 participants and 100 (93%) of 108 participants with at least possible tuberculous meningitis. 31 patients were HIV co-infected (17 [26%] of 65 cases in the Xpert Ultra group and 14 [23%] of 62 cases in the Xpert group; table 1). In HIV-uninfected participants, Xpert Ultra was not more sensitive than Xpert against the reference standard of definite, probable, and possible tuberculous meningitis (p=0·23), nor against the reference standard of definite and probable tuberculous meningitis (p=0·25) or definite tuberculous meningitis (p=0·42; table 3). Both tests were 100% specific in this patient group (table 3).

**Table 3:** Diagnostic performance of Xpert and Xpert Ultra against clinical reference standard, by HIV status

	**Xpert Ultra**		**Xpert**	
	HIV negative	HIV positive	HIV negative	HIV positive
**Reference standard: definite, probable, and possible tuberculous meningitis**				
Positive tests	14/36	9/14	8/35	10/13
Sensitivity	38·9% (24·8–55·1)	64·3% (38·8–83·7)	22·9% (12·1–39·0)	76·9% (49·7–91·8)
Specificity	100·0% (70·1–100·0)	100·0% (43·9–100·0)	100·0% (77·2–100·0)	100·0% (20·7–100·0)
PPV	100·0% (78·5–100·0)	100·0% (70·1–100·0)	100·0% (67·6–100·0)	100·0% (72·2–100·0)
NPV	29·0% (16·1–46·6)	37·5% (13·7–69·4)	32·5% (20·1–48·0)	25·0% (4·6–69·9)
**Reference standard: definite and probable tuberculous meningitis**				
Positive tests	14/29	9/11	8/27	10/12
Sensitivity	48·3% (31·4–65·6)	81·8% (52·3–94·9)	29·6% (15·9–48·5)	83·3% (55·2–95·3)
Specificity	100·0% (80·6–100·0)	100·0% (61·0–100·0)	100·0% (84·5–100·0)	100·0% (34·2–100·0)
PPV	100·0% (78·5–100·0)	100·0% (70·1–100·0)	100·0% (67·6–100·0)	100·0% (72·2–100·0)
NPV	51·6% (24·8–68·0)	75·0% (40·9–92·9)	52·5% (37·5–67·1)	50·0% (15·0–85·0)
**Reference standard: definite tuberculous meningitis**				
Positive tests	14/28	9/11	8/23	10/11
Sensitivity	50·0% (32·6–67·4)	81·8% (52·3–92·9)	34·8% (18·8–55·1)	90·9% (62·3–98·4)
Specificity	100·0% (80·6–100·0)	100·0% (61·0–100·0)	100·0% (84·5–100·0)	100·0% (43·9–100·0)
PPV	100·0% (78·5–100·0)	100·0% (70·1–100·0)	100·0% (67·6–100·0)	100·0% (72·2–100·0)
NPV	53·3% (36·1–69·8)	75·0% (40·9–92·9)	58·3% (42·2–72·9)	75·0% (30·1–95·4)

Xpert Ultra=Xpert MTB/RIF Ultra. Xpert=Xpert MTB/RIF. PPV=positive predictive value. NPV=negative predictive value.

In HIV co-infected participants, the two tests performed similarly against a reference standard of definite and probable tuberculous meningitis (table 3). Against definite, probable, and possible tuberculous meningitis, the sensitivities were 64·3% (95% CI 38·8–83·7) for Xpert Ultra and 76·9% (49·7–91·8) for Xpert (p=0·77; table 3). Against definite and probable tuberculous meningitis, the sensitivities were 81·8% (95% CI 52·3–94·9; nine of 11) for Xpert Ultra and 83·3% (55·2–95·3; ten of 12) for Xpert (p=1·0). Specificities of Xpert Ultra and Xpert were both 100% (table 3). Against a mycobacterial culture reference standard, sensitivities of Xpert Ultra and Xpert in HIV-uninfected participants were 83·3% (55·2–95·3; ten of 12) for Xpert Ultra and 60·0% (31·3–83·2, six of ten) for Xpert (p=0·55), and in HIV co-infected participants sensitivities were 100·0% (95% CI 70·1–100·0; nine of nine) for Xpert Ultra and 100·0% (70·1–100·0; nine of nine) for Xpert (p=1·0).

Xpert can categorise specimen bacterial numbers into high, medium, low, or very low. Xpert Ultra has an additional trace category. The categories obtained from the CSF are shown in the appendix (p 5). The number of samples with medium or low numbers of bacteria were similar between the two groups (ten for Xpert Ultra and 13 for Xpert), suggesting similar baseline bacterial concentrations in the two patient groups. 15 (60%) of 25 CSF samples positive by Xpert Ultra were categorised as containing very low or trace numbers of bacteria compared with eight (38%) of 21 samples with very low bacterial numbers detected by Xpert.

Rifampicin resistance was detected in eight (17%) of 46 positive tests: five (20%) of 25 positive tests by Xpert Ultra and three (14%) of 21 positive tests by Xpert. All five cases categorised as trace positive by Xpert Ultra returned a result of indeterminate resistance. Rifampicin resistance testing was negative in 22 (72%) cases where either Xpert Ultra or Xpert were positive. Of 45 patients with positive CSF MGIT cultures, eight showed rifampicin resistance by phenotypic drug susceptibility testing, all of which were detected by Xpert Ultra (n=5) or Xpert (n=3).

**Figure 3:**
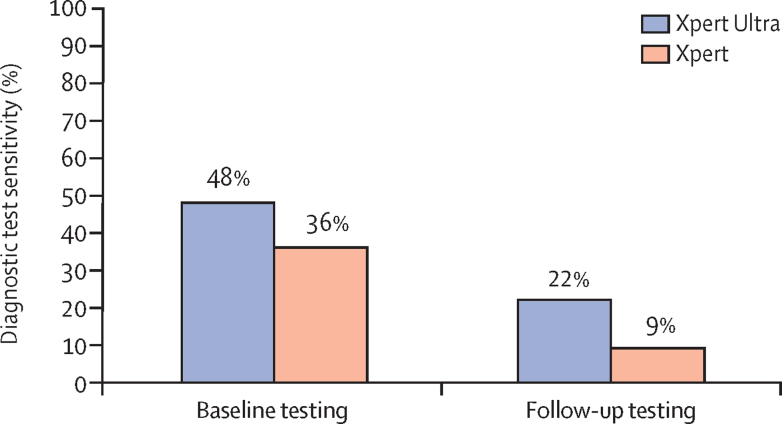
Comparison of the diagnostic sensitivities of Xpert Ultra and Xpert between baseline and testing after 4 weeks of antituberculosis treatment Data shown for 49 patients (27 Xpert Ultra, 22 Xpert) undergoing both baseline and follow-up CSF sampling. All cases with a positive nucleic acid amplification test at follow-up testing (n=8) were positive at baseline testing. Xpert Ultra=Xpert MTB/RIF Ultra. Xpert=Xpert MTB/RIF.

Routine follow-up CSF was sampled and tested in 49 patients treated for tuberculous meningitis (27 by Xpert Ultra and 22 by Xpert). A median of 5·5 mL (IQR 5·0–6·0) CSF was tested in each of the groups. 13 (48%) patients in the Xpert Ultra group and eight (36%) in the Xpert group had a positive test at baseline. After a mean of 27 days (SD 5·9) of antituberculosis treatment in the Xpert Ultra group and 28 days (SD 5·4) in the Xpert group, six (22%) participants in the Xpert Ultra group had a positive test versus two (9%) in the Xpert group (figure 3). Restricting the analysis to those positive by Xpert Ultra or Xpert at baseline, five (38%) of 13 patients in the Xpert Ultra group were still positive after 3–4 weeks' treatment, compared with two (25%) of eight patients in the Xpert group. The influence of drug resistance on a positive test by Xpert Ultra or Xpert at 3–4 weeks after treatment initiation is shown in the appendix (p 3). Median CSF parameters at repeat diagnostic testing in individuals with positive and negative NAATs at repeat testing are also shown in the appendix (p 4), compared with baseline CSF parameters.

## Discussion

In our study, Xpert Ultra was not superior to Xpert for the detection of *M tuberculosis* in CSF of individuals with tuberculous meningitis, using either clinical or culture reference standards. Moreover, Xpert Ultra was not more sensitive than Xpert in HIV-uninfected individuals when compared against all variations of the clinical tuberculous meningitis reference standard or against mycobacterial culture. The sensitivity of both assays was higher in HIV-infected than in HIV-uninfected individuals, probably reflecting the larger numbers of bacteria in CSF samples from these patients.^19^ Additionally, Xpert Ultra appeared to be able to detect more patients with tuberculous meningitis with very low or trace levels of bacteria in their CSF and to be more sensitive than Xpert once antituberculosis treatment had been started.

Previous studies have suggested that Xpert Ultra is significantly more sensitive than Xpert for the diagnosis of tuberculous meningitis.^12^, ^13^ In their cohort of 23 HIV co-infected patients with definite or probable tuberculous meningitis, Bahr and colleagues reported sensitivities of 69·6% for Xpert Ultra and 43·5% for Xpert, compared with 81·8% and 83·3%, respectively, in the HIV co-infected patients included in our study. What might be the explanation for these different results? First, we tested fresh as opposed to stored, frozen CSF samples, which could impair or alter the performance of the assays. Second, we tested the maximum volume of CSF available with one assay rather than dividing the sample in two for simultaneous testing with both assays, which would reduce the number of bacteria available for detection and could reduce the sensitivity of both assays. It is plausible the effect of CSF volume on performance is greater for Xpert than for Xpert Ultra, which can detect trace numbers of bacteria. This possible threshold effect might explain why Xpert Ultra had a higher sensitivity than Xpert for *M tuberculosis* detection in the CSF of HIV-uninfected individuals in our study, albeit without reaching significance, but with no apparent difference between Xpert and Xpert Ultra performance in HIV co-infected tuberculous meningitis. The number of CSF bacteria in HIV-uninfected individuals with tuberculous meningitis could be very close to or below the detection threshold of Xpert but above that of Xpert Ultra, which might drive the difference in performance. HIV co-infected patients have greater numbers of CSF bacteria than do HIV-uninfected patients and, therefore, samples from these patients might exceed the detection thresholds of both assays when large volumes are tested. This CSF bacterial load could account for why test performances in HIV infection appeared more closely aligned than in HIV-uninfected patients. These hypotheses would be supported by showing a correlation between the performance of each assay and CSF volume tested. However, in our study, there was too little variation in the volumes tested to be able to define a correlation with performance of either assay. Therefore, these explanations remain speculative. Finally, the mean copy number of the multicopy amplification target IS*6110* varies between *M tuberculosis* of different lineages,^20^ which might affect Xpert Ultra detection of *M tuberculosis* in different countries. However, isolates belonging to the L2 lineage (the predominant lineage found in Vietnam^21^) have the highest mean copy number of IS*6110*,^20^ which in theory would improve the diagnostic sensitivity of Xpert Ultra at our site, whereas the L4 lineage (predominant in Africa) shows large variation in IS*6110* mean copy number.^22^

The specificity of Xpert Ultra and Xpert in our study was 100% when clinical reference standards were used. The Xpert Ultra specificity decreased slightly only when MGIT culture was used as a reference standard. In a previous study wherein Xpert Ultra was used to test sputum from patients with suspected pulmonary tuberculosis, Xpert Ultra showed reduced specificity compared with Xpert; however, mycobacterial culture was the reference standard used in that study.^11^ A decrease in NAAT specificity is expected when a reference standard of mycobacterial culture is used; culture will only detect viable bacteria, whereas NAAT might detect DNA of dead bacteria that cannot be cultured, leading to apparent false-positive NAAT results against a mycobacterial culture reference standard.^23^

In our study, there were 25 positive Xpert Ultra tests from 55 tested patients with definite, probable, or possible tuberculous meningitis. All 25 had a positive Ziehl–Neelsen smear and 20 (80%) had a positive MGIT. No positive Xpert Ultra results were recorded in patients with a probable or possible diagnosis of tuberculous meningitis, nor in any patient in whom a non-tuberculous meningitis diagnosis was confirmed. In our setting, Xpert Ultra did not diagnose additional cases of tuberculous meningitis missed by other confirmatory mycobacterial testing methods, probably due to the high sensitivity of Ziehl–Neelsen smear microscopy at our site, which has been consistently high over many years.^18^, ^24^ The sensitivity of Ziehl–Neelsen smear at our site repeatedly exceeds that of Xpert and MGIT.^18^ At sites where CSF smear and mycobacterial culture have lower sensitivity, Xpert Ultra could provide more value. At our site, Ziehl–Neelsen smear slides are meticulously examined for 30 min by skilled technicians experienced at identifying acid-fast bacteria in CSF. Centrifugation of CSF at 3000 g for 15 min, resuspension of CSF pellet by vortexing with the sample reagent, and use of 100 μl (20%) of this resuspended CSF pellet for Ziehl–Neelsen smear also improve the diagnostic performance of this test.^25^

The strengths of this study are that it is large, prospective, and randomised, and includes data on the performance of both tests after the start of antituberculosis treatment. The use of randomisation to a single test (ie, with each CSF sample tested by Xpert Ultra or Xpert, not both) is a strength as division of the CSF pellet to allow both Xpert Ultra and Xpert testing to be done on a single CSF sample does not reflect normal clinical practice and could reduce the sample bacillary load and diagnostic sensitivity of both tests. Additionally, laboratory technicians were masked to the patients' clinical characteristics on baseline testing, and testing was done immediately after randomisation. CSF was sampled, processed, and tested in the same way for both Xpert Ultra and Xpert. With the high sensitivity of smear at our site, we were able to microbiologically confirm a high number of tuberculous meningitis cases and, together with MGIT, show that all positive Xpert Ultra tests were true positives.

A limitation of our study is that specificity estimates are mostly from HIV-uninfected individuals; additional data are required to confirm a high specificity of Xpert Ultra in HIV co-infection. Additionally, although individuals with tuberculous meningitis underwent routine HIV testing in our study, HIV testing was not mandatory for all study patients. Another limitation of our study is that, although our study was powered to detect a 25% improvement in diagnostic sensitivity with Xpert Ultra compared with Xpert, we cannot conclude whether Xpert Ultra is superior to Xpert at a lower margin of superiority given our study was not powered to detect smaller differences. Likewise, our study was not powered to detect performance differences in subgroups defined by HIV. Although our randomised study design has strengths, as described above, a limitation with this design is that there is a greater possibility of imbalance between groups than with a design where both diagnostic tests are done on a single CSF sample. There was, however, no evidence of imbalance in any important parameters between the randomisation groups. Additionally, the diagnostic performance of Xpert Ultra compared with Xpert might have been higher than measured in our study because of a higher proportion of individuals randomly assigned to Xpert Ultra than to Xpert having low or trace results.

In summary, the performances of Xpert Ultra and Xpert for the diagnosis of tuberculous meningitis were similar. Although the sensitivity of Xpert Ultra was higher than that of Xpert in patients without HIV infection, this difference was not significant. Our results suggest that Xpert Ultra might perform better than Xpert in patients without HIV infection and remain positive for longer after the start of antituberculosis treatment. Xpert Ultra testing could be preferable when CSF bacillary load is low. However, neither Xpert Ultra nor Xpert have a sufficiently high negative predictive value to rule out tuberculous meningitis. Therefore, the search must continue for a better diagnostic test for tuberculous meningitis.
