# Supplementary material for: Xpert MTB/RIF Ultra versus Xpert MTB/RIF for the diagnosis of tuberculous meningitis: a prospective, randomised, diagnostic accuracy study
Source: Lancet Infect Dis. 2020 Mar;20(3):299–307. doi: 10.1016/S1473-3099(19)30649-8 (PMC7045088; doi:10.1016/S1473-3099(19)30649-8)
Supplement: Supplementary appendix [file mmc1.pdf]

THE LANCET Infectious Diseases

Supplementary webappendix

This webappendix formed part of the original submission and has been peer reviewed. We post it as supplied by the authors.

Supplement to: Donovan J, ThuDDA, Phu NH, et al. Xpert MTB/RIF Ultra versus Xpert MTB/RIF for the diagnosis of tuberculous meningitis: a prospective, randomised, diagnostic accuracy study. *Lancet Infect Dis* 2020; published online Jan 7. https://doi.org/10.1016/S1473-3099(19)30649-8.

Contents

Supplementary results: Diagnostic performance by CSF volume tested 1
Supplementary table 1. Univariable and multivariable analysis of factors predicting microbiological confirmation of tuberculous meningitis 2
Supplementary table 2. Association between TBM drug susceptibility testing and follow up NAAT testing 3
Supplementary table 3. CSF parameters in individuals undergoing repeat diagnostic testing .. 4
Supplementary figure 1. Semi-quantification of positive Ultra and Xpert results 5

Supplementary results: Diagnostic performance by CSF volume tested

CSF volume used for mycobacterial testing was divided into 3 categories; >5mls, 2-5mls and ≤2mls, consistent with CSF volume intervals in a previous study.[18] Diagnostic sensitives of Ultra and Xpert against a reference standard of definite probable and possible TBM were 46·5% (20/43, 95% CI 32·5-61·1%) vs. 36·4% (16/44, 95% CI 23·8-51·1%) for CSF volumes > 5mls, and 40·0% (6/15, 95% CI 19·8-64·3%) vs. 38·5% (5/13, 95% CI 17·7-64·5%) for CSF volumes of 2-5mls. Only three patients with TBM (two definite, one possible) underwent testing of CSF volumes ≤2mls (Ultra; 1/2 positive, Xpert 1/1 positive).

Supplementary table 1. Univariable and multivariable analysis of factors predicting microbiological confirmation of tuberculous meningitis

	Univariable			Multivariable		
**Variable [Q1-Q3]**	**Odds Ratio**	**95% CI**	**p value**	**Odds Ratio**	**95% CI**	**p value**
Age (years) [31, 57]	1·70	1·01-2·89	0·13	1·78	0·95-3·35	0·07
GCS [10, 15]	1·56	0·77-3·16	0·43	0·92	0·21-3·98	0·91
CSF:blood glucose ratio [0·28, 0·61]	0·03	0·004-0·21	< 0·001	0·42	0·16-1·10	0·08
CSF lactate (mmol/L) [2·7, 5·7]	23.9	5·13-111	< 0·001	1·28	0·53-3·11	0·59
Duration of illness [6, 15]	1·83	1·22-2·74	0·01	1·37	0·87-2·16	0·17
CSF protein (g/L) [0·69, 2·3]	4·58	2·04-10·3	0·001	1·93	0·95-3·89	0·07
CSF lymphocyte (%) [37, 87]	0·58	0·29-1·15	0·26	1·17	0·57-2·43	0·66
CSF volume (mls) [5, 6]	1·53	0·88-2·63	0·07	1·37	0·93-2·02	0·11
Female sex [female/ male]	0·30	0·16-0·58	< 0·001	0·32	0·15-0·71	0·01
MRC TBM grade [Grade 1/ Grade 2]	1·48	0·72-3·01	0·53	1·31	0·45-3·80	0·86
MRC TBM grade [Grade 3, Grade 2]	1·01	0·52-1·99	0·53	0·74	0·16-3·52	0·86

Microbiological confirmation of tuberculous meningitis is defined by positive ZN smear, Ultra, Xpert, or MGIT. For age, GCS, CSF:blood glucose ratio, CSF lymphocyte percentage and CSF volume, odds ratios are given for a one unit increase of that variable. For duration of illness, CSF protein and CSF lactate, odds ratios are given for a one unit increase in the log2 of that variable. Quartiles (Q1–Q3) are shown for continuous variables. For categorical variables (sex and MRC TBM Grade) [*x*/*y*] depicts the effect of group *x* to the test positivity rate, relative to group *y*. A ratio greater than 1 corresponds with an increasing prediction of microbiological confirmation of tuberculous meningitis.

CI: confidence interval; CSF: cerebrospinal fluid; GCS: Glasgow coma score; MGIT: mycobacteria growth in tube; MRC: Medical Research Council; Q: quartile; TBM: tuberculous meningitis; Ultra: GeneXpert MTB/RIF Ultra; Xpert: GeneXpert MTB/RIF; ZN: Ziehl-Neelsen

Supplementary table 2. Association between TBM drug susceptibility testing and follow up NAAT testing

NAAT	Baseline CSF NAAT result	Baseline CSF MGIT result	MGIT drug susceptibility testing	Follow up CSF NAAT result	Follow up CSF MGIT result
Ultra	Positive	Positive	RHZES	Positive	Positive
Ultra	Positive	Positive	HZS	Positive	Negative
Ultra	Positive	Positive	S	Positive	Not performed
Ultra	Positive	Negative	Not performed	Positive	Negative
Ultra	Positive	Negative	Not performed	Positive	Negative
Xpert	Positive	Positive	HS	Positive	Negative
Xpert	Positive	Positive	Fully sensitive	Positive	Negative
Ultra	Positive	Positive	RHS	Negative	Negative
Ultra	Positive	Positive	RHS	Negative	Negative
Ultra	Positive	Positive	S	Negative	Negative
Ultra	Positive	Positive	Fully sensitive	Negative	Not performed
Ultra	Positive	Positive	Fully sensitive	Negative	Negative
Ultra	Positive	Positive	Fully sensitive	Negative	Negative
Ultra	Positive	Positive	Fully sensitive	Negative	Negative
Ultra	Positive	Positive	Fully sensitive	Negative	Negative
Xpert	Positive	Positive	HZS	Negative	Negative
Xpert	Positive	Positive	Z	Negative	Contaminated
Xpert	Positive	Positive	Fully sensitive	Negative	Negative
Xpert	Positive	Negative	Not performed	Negative	Negative
Xpert	Positive	Negative	Not performed	Negative	Negative
Xpert	Positive	Positive	Not performed	Negative	Negative

Follow up NAAT testing is shown for samples where baseline NAAT was positive (n=21). Drug susceptibility testing of *Mtb* isolated from CSF is compared with follow up testing results

CSF: cerebrospinal fluid; E: ethambutol; H: isoniazid; MGIT: mycobacteria growth in tube; NAAT: nucleic acid amplification test; R: rifampicin; S: streptomycin; TBM: tuberculous meningitis; Ultra: GeneXpert MTB/RIF Ultra; Xpert: GeneXpert MTB/RIF; Z: pyrazinamide

Supplementary table 3. CSF parameters in individuals undergoing repeat diagnostic testing

	**Ultra**			**Xpert**		
**Median values**	**Baseline**	**Repeat**		**Baseline**	**Repeat**	
	N=25	Positive tests N=6	Negative tests N=19	N=19	Positive tests N=2	Negative tests N=17
WCC (95% CI)	340 207-623	136 63-241	70 42-126	144 104-422	314 240-389	73 35-262
Lymphocyte % (95% CI)	82 28-88	60 45-73	87 84-89	80 67-87	64 64-65	87 67-89
Protein (95% CI)	1.66 1.11-2.75	1.93 1.30-2.38	0.81 0.44-1.52	2.12 1.64-2.88	6.03 5.12-6.93	1.61 0.60-2.41
CSF/blood glucose ratio (95% CI)	0.31 0.20-0.40	0.37 0.32-0.78	0.52 0.45-0.62	0.34 0.25-0.39	0.47 0.40-0.51	0.43 0.40-0.51
Lactate (95% CI)	4.31 3.40-7.60	4.12 3.35-4.67	2.87 2.54-3.40	3.89 3.27-5.50	5.13 4.62-5.63	3.54 3.20-5.19

CI: confidence interval; CSF: cerebrospinal fluid; WCC: white cell count; Ultra: GeneXpert MTB/RIF Ultra; Xpert: GeneXpert MTB/RIF

CSF parameters are shown for 44/49 patients who underwent repeat diagnostic testing (25/27 Ultra, 19/22 Xpert), for whom non-TB CSF parameters were available at both time points. At repeat testing, both Ultra and Xpert CSF parameters were generally worse (elevated WCC, protein and lactate) in those individuals who re-tested as positive, compared with those who re-tested as negative. Compared with baseline testing, CSF parameters of individuals positive by Ultra were generally improved (reduced WCC and lactate, and elevated CSF:blood glucose ratio). Compared with baseline testing, CSF parameters of individuals positive by Xpert were generally worse (elevated WCC, protein and lactate). Whilst the number of cases positive at repeat testing is small, these results may support a proposed role for Ultra in the detection of cases with lower bacillary load (where repeat CSF parameters for cases positive at repeat testing by Ultra are generally improved compared with repeat CSF parameters for cases positive at repeat testing by Xpert).

Supplementary figure 1. Semi-quantification of positive Ultra and Xpert results

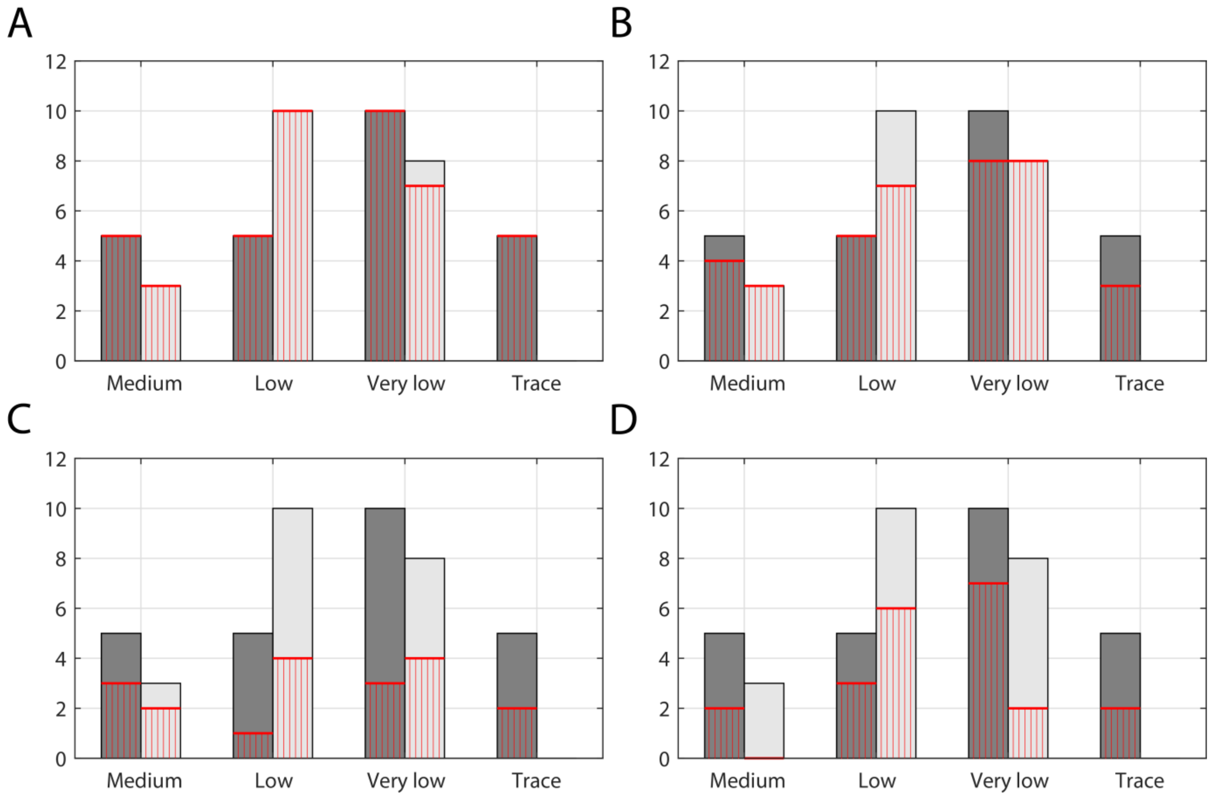

Figure legend. Semi-quantification results for positive Ultra (dark shading, n=25) and Xpert (light shading, n=21). The filled shaded areas represent all positive tests in that semi-quantification category. The patterned (vertical lined) area in each bar represents the number of those cases also positive by ZN smear (**A**), MGIT (**B**), or that have (**C**) or do not have (**D**) HIV co-infection.

Semi-quantification results for Ultra were: medium 5/25 (20·0%); low 5/25 (20·0%); very low 10/25 (40·0%); trace 5/25 (20·0%). Semi-quantification results for Xpert were: medium 3/21 (14·3%); low 10/21 (47·6%); very low 8/21 (38·1%). High bacterial numbers were not present in any of the samples. Ultra: GeneXpert MTB/RIF Ultra; Xpert: GeneXpert MTB/RIF
